# A temperature-adjusted developmental timer for precise embryonic staging

**DOI:** 10.1242/bio.032110

**Published:** 2018-05-17

**Authors:** Konner Winkley, Michael Veeman

**Affiliations:** Division of Biology, Kansas State University, Manhattan, KS 66506, USA

**Keywords:** Arduino, Embryo staging, Thermal time

## Abstract

Developmental biology research depends on careful staging of developing embryos, but the rate of development is extremely sensitive to the temperature at which embryos are raised. It is not always practical to grow embryos at a precisely controlled temperature, so here we describe a simple, inexpensive device based on an Arduino-compatible microprocessor and temperature sensor that provides a metric of developmental time that compensates for changes in temperature. The underlying assumption is that the rate of development will be linear with respect to temperature over an organism's thermal tolerance range. The device measures the ambient temperature and integrates effective degree-minutes over time. For convenience, this is displayed to the user as a temperature-adjusted standard developmental time. In initial testing we have found the device to be extremely helpful for fixing *Ciona* embryos during precise developmental windows.

## Abstract (precis)

**Summary:** We developed and tested a simple electronic device allowing continual temperature monitoring of developing embryos that provides a metric for embryonic staging that compensates for fluctuations in ambient temperature.

## INTRODUCTION

Developmental biologists depend upon staging series to provide a common nomenclature and set of reference points to describe the progression of embryonic development. For poikilotherms (animals that do not regulate their own body temperature), staging series are usually referenced to a standard temperature. The zebrafish staging series, for example, describes the stages of embryonic development at a standard temperature of 28.5°C (Kimmel et al., 1995), whereas the staging series for the ascidian *Ciona* describes development at 18°C (Hotta et al., 2007). Development typically occurs normally, however, over a broader thermal tolerance range. Experimenters will sometimes exploit this to speed up or slow down development for experimental convenience. Growing embryos at precisely controlled temperatures can be surprisingly challenging. Room temperatures fluctuate considerably in most buildings. Temperature controlled incubators are often imperfectly adjusted or calibrated, and are subject to fluctuations from the heater or cooler cycling on and off, or the door being opened and shut. Experiments also frequently require embryos to be removed from incubators periodically for various manipulations.

The inspiration for the device developed here came from our lab's ongoing research on notochord morphogenesis in the simple ascidian chordate *Ciona* (Reeves et al., 2014, 2017; Carlson et al., 2015). We frequently found ourselves collecting closely spaced time series of embryos so as to be able to fix, stain and image embryos at very specific points in development such as precise stages of cell cycles in specific blastomeres. These precise stages can only be distinguished after embryos have been fixed and imaged by confocal microscopy. *Ciona* development is highly stereotyped, but we found that we were often missing the desired stages because our embryology room was slightly cooler or warmer than usual. Rearing embryos in temperature controlled incubators is helpful, but even there the eggs/embryos have to be out of the incubator for varying amounts of time to fertilize and dechorionate them, sort out embryos with normal development, fix time-points and other experimental manipulations.

We hypothesized that constant temperature monitoring would allow much more accurate staging of developing embryos. For most poikilothermic model organisms, the rate of development is thought to vary approximately linearly with respect to temperature within the organism's thermal tolerance range (Trudgill et al., 2005; Damos and Savopoulou-Soultani, 2012). Various temperature loggers are commercially available that allow temperature to be recorded over time and retrospectively analyzed, but we were not able to identify any that could be configured or programed to show a running measure of temperature integrated over time or any related metric that would be useful in staging embryos grown under fluctuating temperatures. It occurred to us, however, that such a device could be easily built and programed using an Arduino-compatible microcontroller connected to a temperature probe and a small display.

## Relationship between temperature and developmental rate

The influence of temperature upon the rate of development has been widely investigated in both plant and animal systems. Experimenters have generally found that, within an organism's thermal tolerance range, there is a linear relationship between the rate of development (the reciprocal of the length of time it takes to achieve some developmental landmark) and temperature (reviewed by Trudgill et al., 2005; Damos and Savopoulou-Soultani, 2012). This linear relationship can be represented by an equation in the form r=aT+b where T is temperature, a gives the slope and b the y-intercept. The x-intercept of this line is –b/a and it represents the theoretical temperature at which the velocity of development drops to 0. The length of time (D) it takes to reach a given developmental landmark can then be represented as D=K/(T-T_0_) where T is the actual temperature, T_0_ is the developmental zero temperature and K is a thermal time constant (degree-minutes above T_0_ needed to reach the landmark). The relationship between developmental time at temperature T and a reference temperature T_ref_ can thus be represented as D_ref_=D(T-T_0_)/(T_ref_-T_0_).

It follows that the only species-specific parameter needed to relate developmental time to a reference temperature is the species’ developmental zero temperature T_0_. This can be estimated by growing embryos at multiple temperatures, plotting the rate of development as a function of temperature, and then finding the x-intercept of the linear regression line. T_0_ values have been published for many species, but not for *Ciona*. The *Ciona* staging series paper, however, contains information on the time it takes to reach key stages at several different temperatures (Hotta et al., 2007). We were able to use this data to estimate that the T_0_ for the Japanese population of *Ciona* studied in that paper is ∼1°C. We made this estimate based on a linear regression of the reciprocal of the time it took for embryos grown at four different temperatures to reach Stage 19, using data extracted from Supplemental Fig. 1 in Hotta et al.'s paper. Our lab uses *Ciona* from the much warmer San Diego bay area and we have found that their T_0_ seems to be considerably higher at ∼7.5°C (data not shown). This parameter may need to be optimized for different populations, especially if they are wild-caught populations from different climates. The thermal biology of *Ciona* embryonic development is underexplored, but wild populations are known to span a broad range of temperatures (Bouchemousse et al., 2016a).

## Design and construction

The Arduino platform and integrated development environment allows scientists such as ourselves with minimal electronics experience to build and program simple microcontroller-based devices (Pearce, 2012). Our guiding principle in designing the Temperature-Adjusted Developmental Timer (TADT) device was that it should require a minimum of soldering and be straightforward to build, making it as easy as possible for both ourselves and other developmental biology labs to build their own instruments.

We chose the Arduino-compatible Feather board from Adafruit (New York, USA) as the heart of the device because it is smaller than a standard Arduino UNO but has an integrated lithium-polymer (LiPo) battery controller and a good ecosystem of commercially available add-on boards (‘FeatherWings’) including an excellent OLED display. We used a DS18B20 digital temperature sensor because it is relatively precise and accurate, inexpensive, and easy to connect to an Arduino. We also chose the Adafruit Terminal Block Wing because it provides a straightforward way to connect the temperature sensor wiring and incorporate a needed pull-up resistor with a minimum of soldering.

The bill of materials is shown in Table 1, and the entire device can be built for only US$80 as of June 2017. Step by step assembly instructions are as follows:

**Table 1.**
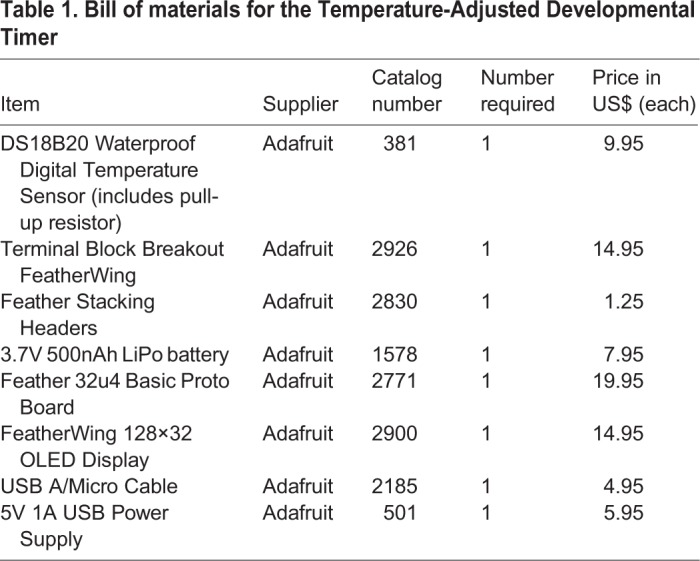
**Bill of materials for the Temperature-Adjusted Developmental Timer**

**Fig. 1.**
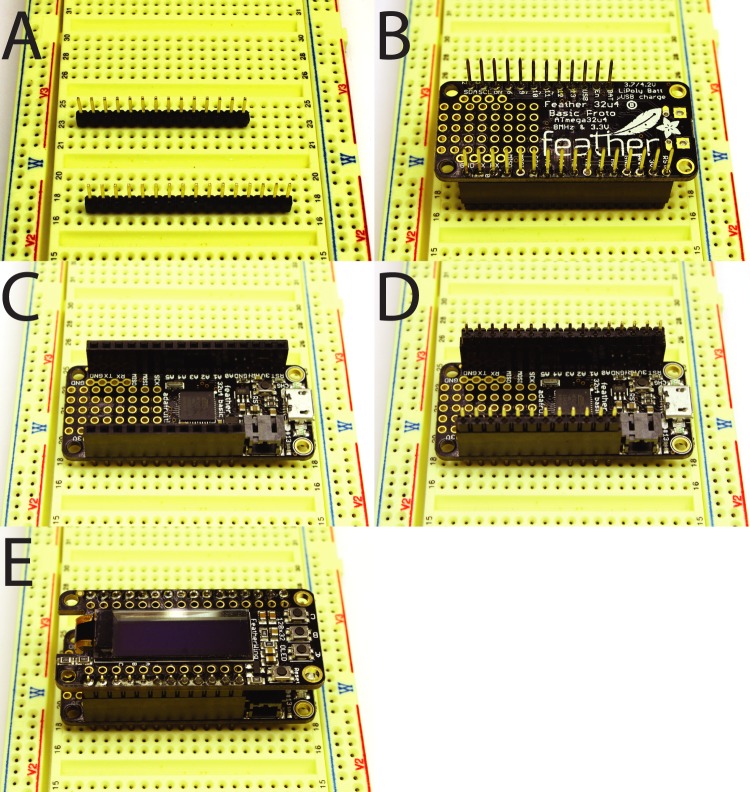
**Soldering header strips.** (A) Non-stacking headers inserted into a solderless breadboard serving as a base for soldering. (B) Stacking headers soldered into place on the Feather board. (C) Stacking headers and Feather board flipped so that the male ends of the stacking headers can be placed into the solderless breadboard. (D) Long ends of the headers from OLED display placed into female ends of stacking headers attached to the feather board. (E) OLED display placed onto headers and soldered into place.
2) Insert the long sides of the headers from the OLED display into the female side of the stacking headers now soldered onto the Feather (one set will need to be trimmed to the correct length). Place the OLED display on top of these and solder the headers into place (Fig. 1C-E).
3) Solder a short piece of 22AWG hook-up wire and a 4.7K ohm resistor onto the Terminal Block Wing as shown in Fig. 2A,B. This acts as a pull-up resistor between the 3.3v power source and general purpose input/output pin 5 (GPIO5).

1) Solder stacking headers onto the Feather board (they can be easily held in place for soldering by using a pair of non-stacking headers inserted into a solderless breadboard) (Fig. 1A,B).

**Fig. 2.**
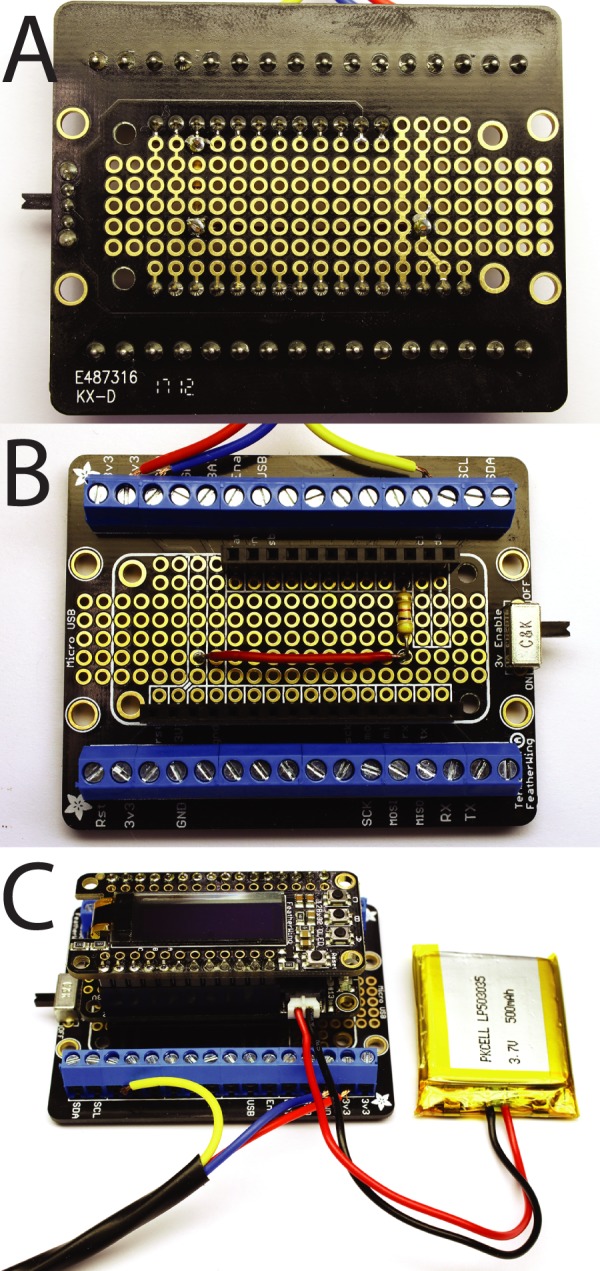
**Assembling the TADT.** (A,B) Top (A) and bottom (B) view of terminal breakout board with stacking headers and pull-up resistor soldered into place. (C) Completed device with Feather board attached to terminal breakout board and LiPo battery.
4) Connect the wires from the temperature sensor to the terminal blocks as shown in Fig. 2B,C. The red wire connects to 3v power, the blue wire to ground and the yellow wire to GPIO5.
5) Stack the Feather board on top of the Terminal Block Wing and the OLED wing on top of the Feather (Figs 2 and 3).

**Fig. 3.**
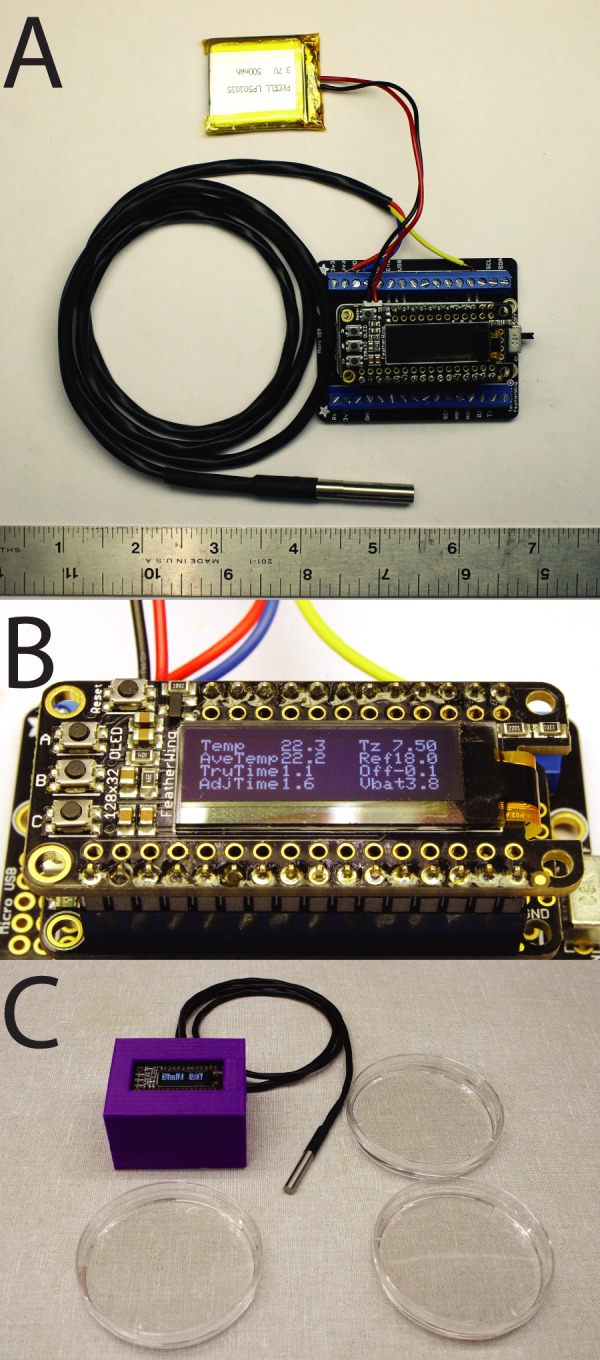
**Assembled TADT device.** (A) TADT device with temperature probe and LiPo battery connected. Ruler for scale is in inches. (B) Device display when powered on. (C) TADT in use with temperature probe in close proximity to 90 mm embryo culture dishes.
6) Connect the LiPo battery to the JST connector on the Feather board. (The battery is optional. The device will work well without it as long as it is connected to a USB power source. The battery provides a measure of convenience in case of a power outage or accidental disconnection, or if it has to be moved farther than the USB cable can reach. The battery and charging circuit have extensive safety features, but there are fire hazards associated with lithium batteries and builders should make their own decisions as to whether battery power is appropriate.)

### Enclosure

**Fig. 4.**
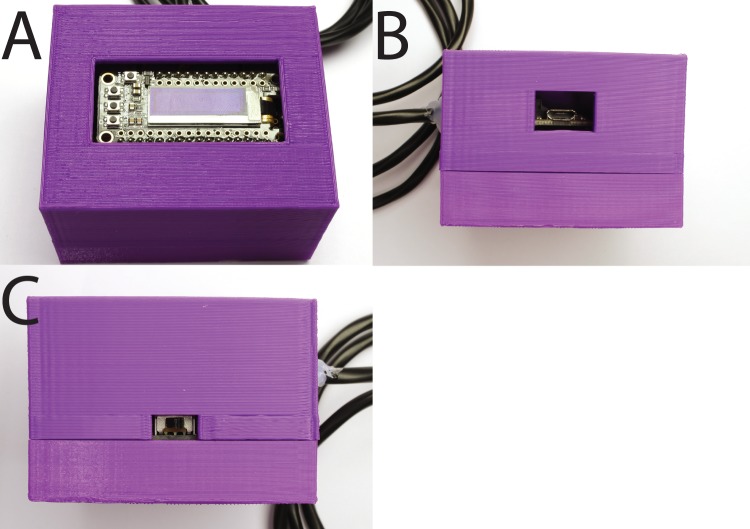
**3D printed enclosure.** (A) Display opening. (B) Micro-USB port opening. Temperature probe pass-through can be seen on the left side. (C) Power switch opening. Temperature probe pass-through can be seen on the right side.

The device can be used as is, but it is desirable to house it in an enclosure to protect it and give it a more finished appearance. We designed a two-piece enclosure suitable for 3D printing (Fig. 4). The enclosure consisted of a lower base piece (base.stl, downloadable from https://github.com/chordmorph/TADT) and an upper lid (lid.stl, downloadable from https://github.com/chordmorph/TADT) and can be printed using inexpensive polylactic acid (PLA) filament in a hobby-style 3D printer; in our case a Makerbot 5 at the KSU Hale library. The base piece has mounting points which align to the mounting holes on the breakout board, allowing it to be screwed solidly in place. There are holes in the case for the temperature probe cable and to allow access to the power switch, the micro-USB port, and the display.

Alternatively, the TADT can be made to fit relatively easily in an inexpensive model 7593K26 enclosure from McMaster-Carr. We cut an opening in the top to fit the OLED display and openings in the side to pass the temperature and USB cables into the enclosure and allow access to the on/off switch. A scrap piece of ¼″ (5 mm) acrylic was cut to use as a spacer to raise the top of the display close to flush with the opening. The spacer and protowing were then mounted semi-permanently into the case bottom with hot melt glue (not shown).

### Software

The program (‘sketch’ in Arduino terminology) we have written to control the device is conceptually very simple (TADT.ino, downloadable from https://github.com/chordmorph/TADT). Every second, it polls the temperature probe to determine the current temperature. It then keeps a cumulative tally of degree-minutes above T_0_ (for implementation reasons it is actually degree-milliseconds since the sketch started running). This cumulative effective temperature can be converted back into a measure of time corrected to a reference temperature by dividing by T_ref_-T_0_. The sketch then uses the OLED display to show four different experimental values of interest including the current temperature, the mean temperature over the course of the experiment, the time elapsed since the sketch started and the temperature-adjusted time elapsed. As a convenience, it also displays the battery voltage as an indication of charge status, as well as the T_0_ and T_ref_ values and an optional offset value used to calibrate the temperature sensor (Fig. 3B).

The sketch depends on several libraries, including the Onewire library to connect to the DS18B20 temperature sensor using the Dallas/Maxim 1-wire protocol, the Adafruit_GFX base library for displaying text and graphics on various small displays and the Adafruit_SSD1306 library that adds specific support for the 128×32 OLED display and driver chip used in the OLED FeatherWing. The Onewire library only provides very basic support for communicating with 1-wire devices, and additional code is needed to actually power the sensor and obtain temperature readings. We used a function obtained online from bildr.org that is simple and well documented (https://github.com/bildr-org/DS18B20, http://bildr.org/2011/07/ds18b20-arduino/). Excellent instructions for configuring and using the Feather board are available from Adafruit (https://learn.adafruit.com/adafruit-feather-32u4-basic-proto/arduino-ide-setup).

1) Download the Arduino IDE from www.arduino.cc.
2) Open it, go to File>Preferences>Additional Boards Manager URLs and add https://adafruit.github.io/arduino-board-index/package_adafruit_index.json to the dialog box.
3) Tools>Boards>Board Manager select ‘Adafruit AVR Boards’ to add support for the Feather 32u4.
4) Close and reopen the IDE and go to Tools>Boards>Feather 32u4.
5) Go to Sketch>Include Library>Manage Library.
6) Use the library manager to install the following libraries:
  - Onewire (used for communicating with the DS18B20 temperature sensor)
  - Adafruit_GFX (base library for displaying text and graphics)
  - Adafruit_SSD1306 (adds specific support for the 128×32 OLED)
7) Copy the TADT sketch (https://github.com/chordmorph/TADT/blob/master/TADT.ino) into your Arduino sketchbook folder (likely/Documents/Arduino).
8) Open the TADT sketch in the IDE.
9) Connect the device to the computer with a suitable USB cable.
10) Once the Feather board has been recognized by the IDE, it should become visible and can be selected under Tools>Port.
11) Upload the sketch to the board by clicking the upload button in the sketch window.

### Configuring the device

There are three parameters that can be changed in the sketch. Two of these are constants for the linear degree minute model, including the developmental zero temperature T_0_ and the reference temperature T_ref_. These are set by default to 7.5°C and 18.0°C for *Ciona*, but can be changed in the sketch and then re-uploaded to the device. There is also an offset value that can be adjusted to calibrate the DS18B20 temperature sensor (set to 0°C by default). An easy test can be performed by swirling the temperature sensor in an ice/water bath. If the sensor reads above 0°C, then a negative offset can be used to correct it and vice-versa. The DS18B20 is specified as being accurate to within±0.5°C, but of the five sensors we have tested, we have found three of them to be within ∼0.1°C of 0 in an ice/water bath. The other two were both more than half a degree off and behaved unpredictably across changing temperatures. The temperature sensor is inexpensive (US$10) so we suggest buying a few more than needed and discarding any defective ones.

### Using the device

The TADT device will begin measuring the temperature and calculating temperature-adjusted minutes as soon as it is powered up using the switch on the terminal block wing. The temperature probe should be kept as near as possible to the embryos of interest (Fig. 3C). The device can be reset using the reset button on the top left of the display. We usually turn the device on and let the temperature probe equilibrate with the surroundings prior to fertilizing embryos, and then hit the reset button immediately after adding sperm to the eggs. The other three buttons on the display board are not used in the current implementation. For long-term use, it is best to keep it attached to a USB power source, but the LiPo battery (if used) will power it for several hours. The battery voltage will read ∼4.2v when fully charged and drop to ∼3.4v when nearly discharged.

### Biological validation

**Fig. 5.**
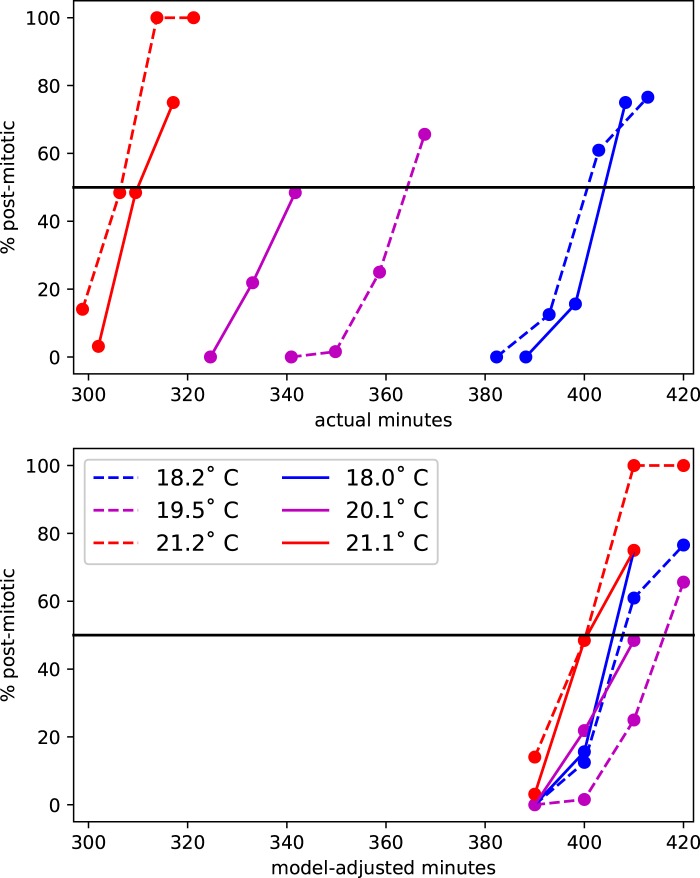
**Biological validation.** Timing of the final primary notochord cell division (percent of cells that have completed mitosis) for *Ciona* embryos grown at different temperatures. The top panel shows actual time. The bottom panel shows model-adjusted time using TADT devices with parameters T_0_=7.5°C and T_ref_=18°C. The two separate biological replicates are represented with solid versus dashed lines. The legend applies to both panels.

In initial trials we estimated that the T_0_ of our *Ciona* collected from San Diego area marinas was ∼7.5°C, and that the final cell division in the primary notochord lineage takes place at approximately 400 min post-fertilization at the reference temperature of 18°C. To test the TADT, we grew dechorionated sibling embryos from a single fertilization in two different incubators set to approximately 18°C and 20°C, as well as on the benchtop at approximately 21°C. We used three different TADTs to simultaneously monitor each group of embryos and found that the actual mean temperature of each condition was approximately 18.0°C, 20.1°C and 21.1°C. We repeated the experiment, this time with mean temperatures of 18.2°C, 19.5°C and 21.2°C. For each temperature/replicate, we fixed embryos at 390, 400 and 410 adjusted minutes (for some replicates we also collected a 420 adjusted minute time-point). For each batch of fixed embryos, we also recorded the time elapsed in actual minutes. We stained the embryos and imaged four each at each time-point/temperature/replicate by confocal microscopy. We then scored each of the 16 primary notochord precursor cells per embryo for whether it was premitotic, mitotic or postmitotic with respect to the final notochord cell division. As shown in Fig. 5, the timing of this cell division is closely aligned between the different temperatures when measured in temperature-adjusted minutes despite being widely separated in real time.

## DISCUSSION

We initially built a prototype device using an Arduino with an LCD display, and have since built three ‘production’ devices using Feather 32u4 boards. The hardware and software are both very simple, and it would be easy to implement a similar concept using many different processor boards, temperature sensors and displays. Only two parameters in the code need to be changed to adjust the reference temperature and developmental zero temperature. We also include a software calibration parameter that can be used to adjust for slight differences between different DS18B20 temperature sensors. The software can easily be customized to display different measurements of interest. One could, for example, directly display integrated degree-minutes, convert the data into a percentile score (e.g. 17% of normal development) or provide a continuous estimate mapped onto a discrete staging series (e.g. Stage 3.35).

The TADT works well in our hands, but we anticipate several potential improvements. The DSB18B20 digital temperature sensor could be replaced with a more accurate and precise platinum thermocouple. It would also be relatively straightforward to integrate a data logging capability to record a detailed temperature trajectory for each experiment. Adafruit sells a version of the Feather with an integrated SD card holder that would work well for this.

Another potential improvement would be to incorporate a more sophisticated model of the relationship between temperature and the rate of development. The linear degree-minute model used here was effective for *Ciona* over the timeframe tested, but more sophisticated non-linear models have been developed in other contexts (Schoolfield et al., 1981; Shi et al., 2011).

Our use case here was to help fix embryos during very precise developmental windows, but there are many contexts in which thermal time would provide a fine-grained and inherently temperature-compensated staging metric for developmental biology research. More work will be needed to determine the variation in T_0_ across different populations of *Ciona* and to test whether a linear model is appropriate across a broader range of stages and temperatures, but the concept appears broadly valid and the device is easily configurable for other model organisms. A potential complication for wild-caught animals as used by most *Ciona* labs is that there is considerable seasonal variation in ocean temperature and there may be seasonal plasticity in thermal biology. We have not yet addressed this issue, but the TADT provides a framework for doing so. Modern hobbyist ‘maker-movement’ microcontrollers make custom instrumentation of this sort increasingly accessible.

## MATERIALS AND METHODS

Adult *Ciona intestinalis* Type A (recently renamed *Ciona robusta*) (Pennati et al., 2015; Bouchemousse et al., 2016b) were obtained from Marine Research and Educational Products (San Diego, USA). Fertilizations and dechorionations used standard methods (Veeman et al., 2011). Embryos were fixed in 2% paraformaldehyde in seawater and stained with Alexa488 phalloidin (Invitrogen). Confocal z-stacks were collected on a Zeiss 880 LSCM using a 40× 1.3NA objective. Primary notochord cells were manually scored for cell cycle stage based on tissue morphology and the presence/absence of a distinct nucleus.
